# Tungsten Alloy and Cancer in Rats: Kalinich Responds

**Published:** 2005-12

**Authors:** John F. Kalinich

**Affiliations:** Armed Forces Radiobiology, Research Institute, Bethesda, Maryland, E-mail: kalinich@afrri.usuhs.mil

We would like to address Schell’s comments about our article published in *EHP* (Kalinich et al. 2005). Schell expresses concern about certain statements we made in our article about embedded tungsten alloy fragments, especially our reference to the undisputed Centers for Disease Control and Prevention (CDC) finding that there is an increased incidence of childhood leukemia in areas where there are high levels of environmental tungsten (CDC 2003; Sheppard and Witten 2004). Schell contends that our results showing the carcinogenic potential of embedded tungsten alloy fragments have no bearing on the situation in Fallon, Nevada, and believes that our mentioning them “is both inappropriate and misleading.” We respectfully disagree.

In our article (Kalinich et al. 2005) we report an unexpected response in rats to tungsten alloys that could not have been predicted by looking at tungsten toxicity alone. We suggested that our results support the advisability for further consideration of tungsten compounds or synergistic effects of tungsten with other environmental factors in cases such as Fallon. We cited several reports in support of such a view. Miller et al. (2001, 2002) indicated that the presence of tungsten in an *in vitro* model system increased the toxicity of both nickel and cobalt in a synergistic manner. Wei et al. (1985, 1987) reported that tungsten exhibited a promoting effect on *N*-nitroso-*N*-methylurea–induced mammary carcinogenesis in rats. Other investigators have also suggested the cause for the Fallon cancer cluster might be an as yet uninvestigated factor or the result of simultaneous or sequential exposure to one or more agents (Daughton 2005).

At this time it is not clear whether these similar findings from diverse research are an unrelated coincidence or whether they suggest a toxicologic property of tungsten not yet understood. What is clear, however, is the need for further research in this area, not only a toxicologic assessment of tungsten alone but also potential synergistic interactions with known toxic agents. The currently proposed National Toxicology Program study of tungsten is an important first step in resolving these issues.
